# Association of physical fitness components and health-related quality of life in women with systemic lupus erythematosus with mild disease activity

**DOI:** 10.1371/journal.pone.0212436

**Published:** 2019-02-20

**Authors:** Blanca Gavilán-Carrera, Jaqueline Garcia da Silva, José A. Vargas-Hitos, José M. Sabio, Pablo Morillas-de-Laguno, Raquel Rios-Fernández, Manuel Delgado-Fernández, Alberto Soriano-Maldonado

**Affiliations:** 1 Department of Physical Education and Sport, Faculty of Sport Sciences, University of Granada, Granada, Spain; 2 Department of Personality, Assessment, and Psychological Treatment, School of Psychology, University of Granada, Granada, Spain; 3 Systemic Autoimmune Diseases Unit, Department of Internal Medicine, “Virgen de las Nieves” University Hospital, Granada, Spain; 4 Systemic Autoimmune Disease Unit, Department of Internal Medicine, “San Cecilio” University Hospital, Granada, Spain; 5 Department of Education, Faculty of Education Sciences, University of Almería, Almería, Spain; 6 SPORT Research Group (CTS-1024), CERNEP Research Center, University of Almería, Almería, Spain; Campus Bio-Medico University of Roma, ITALY

## Abstract

**Objectives:**

To study the association of different components of physical fitness [flexibility, muscle strength and cardiorespiratory fitness (CRF)] and a clustered fitness score with health-related quality of life (HRQoL) in women with systemic lupus erythematosus (SLE) and to analyze whether participants with high fitness level have better HRQoL.

**Methods:**

This cross-sectional study included 70 women with SLE (aged 42.5; SD 13.9 years). The back-scratch test assessed flexibility, the 30-sec chair stand and handgrip strength tests assessed muscle strength, and the 6-min walk test (*n* = 49) assessed CRF. HRQoL was assessed through the 36-item Short-Form Health Survey (SF-36).

**Results:**

Flexibility was positively associated with the physical function dimension and the physical component summary (PCS) (r_partial_ between 0.26 and 0.31; *p*<0.05), and negatively related with social functioning dimension (r_partial_ = -0.26; *p*<0.05). Muscle strength was positively associated with the physical function, physical role, bodily pain dimensions and the PCS (r_partial_ between 0.27 and 0.49; all *p*<0.05). CRF was positively associated with the physical function and bodily pain dimensions, and PCS (r_partial_ between 0.39 and 0.65; all *p*<0.05). The clustered fitness score was associated with the physical function (*B* = 17.16) and bodily pain (*B* = 14.35) dimensions, and the PCS (*B* = 6.02), all *p*<0.005. Patients with high fitness level had greater scores in the physical function, physical role, and bodily pain dimensions and the PCS, all *p*≤0.05.

**Conclusions:**

Our study suggests that muscle strength and CRF are positively associated with HRQoL, while flexibility showed contradictory results. These findings highlight the importance of maintaining adequate fitness levels in women with SLE.

## Introduction

Systemic lupus erythematosus (SLE) is an autoimmune disease that may involve any organ or system and produce a wide spectrum of clinical manifestations [1]. The goals of SLE therapy are reducing disease activity, preventing organ damage and improving health-related quality-of-life (HRQoL) [2]. Although the spectrum of treatment options and the general knowledge of SLE have improved in recent decades (i.e. leading to similar survivals rates than the general population) HRQoL continues to be compromised in this population [3]. Evidence shows that patients with SLE present a clearly deteriorated HRQoL not only compared to healthy individuals [4–7] but also to other chronic diseases [8].

The determinants of HRQoL in SLE are complex but may include factors related to disease and treatment, health care provision [1], or psychological variables [9]. In addition to medical therapy, non-pharmacological therapies, such as exercise, seems to be an efficient and safe alternative to improve physical fitness [10], symptomatology and HRQoL of patients with SLE [11,12]. Aerobic training [13–18], as well as strength and flexibility training [13,18,19] have shown a variable efficacy in improving diverse dimensions of HRQoL in SLE. Noteworthy, exercise interventions are typically poorly described and provide insufficient information to warrant replication and a clear understanding on how the different components of physical fitness must be trained along an intervention period for SLE. Therefore, the optimal exercise intervention for SLE is still unclear [12].

Physical fitness is a strong health marker in the general population [20] and in other rheumatologic diseases including rheumatoid arthritis [21] and fibromyalgia [22–24], among others. It has been reported that patients with SLE present a reduced cardiorespiratory fitness (CRF) [4–7,25], muscular strength and functional capacity [26]. Functional aerobic impairment [6,25] and low levels of strength [26] in SLE are negatively correlated with perception of severity [6], fatigue [6,26], and age-related arterial stiffness [25]. Furthermore, a fitness level below the criterion-referenced standards could lead to a premature loss of independence [27]. In regards to HRQoL, this outcome has been also positively related to higher CRF [7,28] and muscle strength in SLE [26,29,30]. However, the scope of previous research is generally limited to physical dimensions ignoring the also relevant social and mental spheres of HRQoL [31,32]. As patient-reported outcomes are considered key variables in the assessment and management of patients with SLE[33], and fitness might be enhanced through exercise interventions [10], a more comprehensive characterization of the association of the different dimensions of HRQoL with physical fitness might inform the design of randomized trials and the development of therapeutic exercise protocols for this population.

Therefore, the aims of this study were i) to examine the association of different components of physical fitness [flexibility, muscle strength and CRF] and a clustered fitness score with HRQoL in women with SLE and ii) to analyze whether participants with high fitness level have better HRQoL. We hypothesize that i) all physical fitness components, as well as the clustered fitness score, will be positively related to different physical, social and mental dimensions of HRQoL and that ii) participants with high fitness level have better HRQoL.

## Materials and methods

### Design and participants

For this cross-sectional study, a total of 172 patients with SLE were invited to participate in the study through the Systemic Autoimmune Diseases Unit of the “Virgen de las Nieves” University Hospital and the “San Cecilio” University Hospital (Granada, Spain). After providing detailed information about the aims and study procedures, participants provided written informed consent. The inclusion criteria were: i) women aged 18–70 years), ii) with ≥4 SLE classification criteria according to the American College of Rheumatology [34], and iii) with a minimum medical follow-up of 1 year at the units and presenting clinical stability, defined as no changes in the systemic lupus erythematosus disease activity index (SLEDAI) and/or the treatment during 6 months before the study. Exclusion criteria were as follows: not being able to read, understand and/or sign the informed consent; presence of concurrent fibromyalgia; history of clinical cardiovascular disease in the last year, receiving a biological treatment or required doses of prednisone (or equivalent) greater than 10 mg/day in the last 6 months. The Research Ethics Committee of Granada reviewed and approved the study protocol.

The patients were evaluated on a single day at the “Virgen de las Nieves” University Hospital, where sociodemographic data and clinical history were obtained, anthropometric measures and body composition were assessed, and the fitness tests were carried out. Participants were instructed in how to fill the questionnaires related to HRQoL, depression, and fatigue at home and returned them throughout the same week. The questionnaires were collected and checked by the research team for potential non-response.

### Measurements

#### Health-related quality of life (HRQoL)

The HRQoL was assessed with the Spanish version of the Short-form 36 health survey questionnaire (SF-36) [31], which is validated for patients with SLE [32]. The SF-36 questionnaire contains 36 items grouped into 8 dimensions: physical function, physical role, bodily pain, general health, vitality, social functioning, emotional role and mental health. The scores range from 0 to 100, where higher scores indicate better health. The physical component summary (PCS; range 0–100) and the mental component summary (MCS; range 0–100) were also calculated.

#### Physical fitness components

Physical fitness components were assessed with the standardised Senior Fitness Test Battery [35] plus the handgrip strength test [36]. Upper-body flexibility was assessed with the *back scratch test*, where the cm between (positive distance) or overlap (negative distance) of the middle fingers behind the back was recorded twice for each arm. The best scores of dominant, and non-dominant side were used and the total mean was calculated. CRF was assessed with the *6-min walk test* that measures the distance in meters that a participant is able to walk along a 45.7 m course within 6 minutes. Lower-body muscle strength was assessed with the *30-sec chair stand test*, in which we registered the times an individual is able to rise, from a sitting position to a full stand position with arms folded across chest. Upper-body muscle strength was assessed with the *handgrip strength test* using a digital dynamometer (TKK 5101 Grip-D; Takey, Tokyo, Japan), as described elsewhere [36]. Participants performed the test twice (alternately with both 2 hands), with 1 min of rest between trials. The best score from both hands (dominant and non-dominant) were averaged and the total mean was calculated.

#### Anthropometry and body composition

Height was measured in cm using a stadiometer (SECA 222, Hamburg, Germany), and weight in kg with a bioimpedance device (InBody R20, Biospace, Seoul, Korea). The body mass index (BMI, kg/m^2^) was calculated.

#### Depression

The Beck Depression Inventory-second edition (BDI-II) [37] is a 21-item self-report measure designed to assess depressive symptomatology. Each depressive symptom is rated from 0 (not present) to 3 (severe) according to how patients felt during the past 2 weeks. The BDI-II provides a single overall score (0–63) where higher score represents higher depressive symptomatology.

#### Fatigue

Fatigue severity was measured with the Spanish version of the Multidimensional Fatigue Inventory (MFI-S) that contains 5 scales. Each subscale consists of four items ranging from 4 to 20, with higher scores indicating greater fatigue. For the present study, only general fatigue dimension was included.

#### Sociodemographic variables and clinical history

All participants filled out a socio-demographic and clinical data questionnaire to collect information including age, educational level, occupational status, and SLE data (diagnostic criteria, year of diagnosis, time of evolution and treatments). The disease activity was measured by the SLEDAI [38].

### Statistical analysis

Descriptive statistics were performed to examine sociodemographic and clinical characteristics of the sample. The continuous variables were presented as mean and standard deviation, and categorical variables with frequencies and percentages. In addition, we calculated the percentage of patients achieving the fitness standards proposed by Rikli and Jones [27] for the *Senior Fitness Test Battery* to maintain physical independence in later years. The values recommended for the lowest range of age (women between 60 and 64 years) of the fitness tests included in this study (*30-sec chair stand* and *6-min walk*) were used. An independent t-test and χ2 test were used to compare sociodemographic and clinical features between participants with low and high fitness level.

To study the association between fitness and HRQoL, different approaches were conducted. First, partial correlations were used to assess the association of the different components of physical fitness with all dimensions of the SF-36 questionnaire while accounting for the following covariates: age, BMI, BDI-II total score and MFI general fatigue score. Because disease activity (SLEDAI) [39,40], systemic damage index (SDI) and disease duration (according to preliminary analyses) could be potentially related with HRQoL, additional adjustments for these variables were included in all the analyses. Next, a clustered fitness score was calculated as the weighted average (taking into account the number of tests assessing each component) of flexibility, muscle strength and CRF z-scores [(value-mean)/SD]. The association between the clustered fitness score (as a global measure of fitness including all components) and HRQoL was assessed with linear regressions analyses. All dimensions of the SF-36 questionnaire were entered in separate regression models as dependent variables and the clustered fitness score along with the covariates were introduced as independent variables using the ‘enter’ method.

Finally, for those dimensions of HRQoL significantly associated with fitness components in prior correlations analyses, further analysis of covariance (ANCOVA) compared the differences in SF-36 scores between patients with low and high fitness level. The median value of the sample for each fitness test [flexibility (1.35 cm), upper-body muscle strength (24.28 kg/cm^2^), lower muscle strength (15 repetitions) and CRF (575 meters)] was used to consider high (≥median value) or low (<median value) fitness.

The analyses were performed using Software SPSS version 21.0 and the statistical significance was set at p≤0.05.

## Results

**Fig 1:**
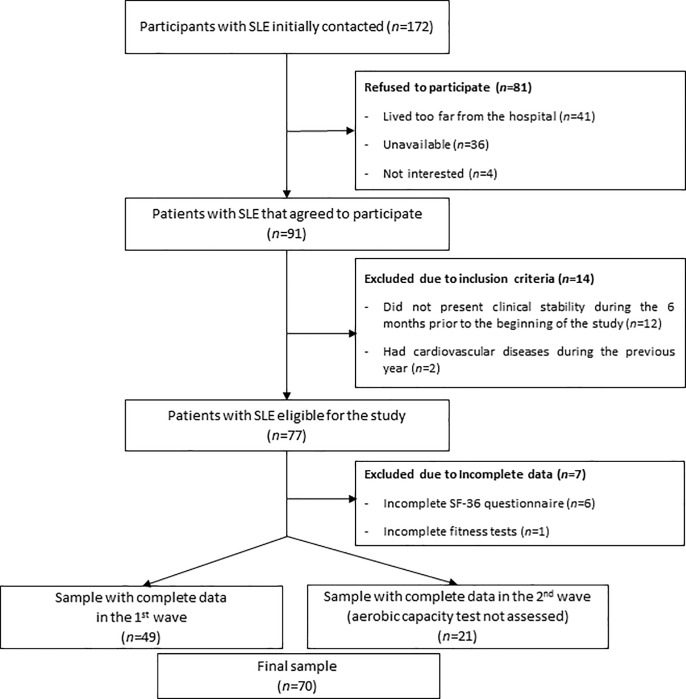
Flow diagram of inclusion of women with systemic lupus erythematosus (SLE) for the present study. SF-36: 36-item Short-Form Health Survey.

The flow diagram of the participants included in this study is presented in Fig 1. A total of 77 patients met the inclusion criteria, agreed to participate and were assessed in 2 waves (49 women were assessed in October 2016 and 28 were assessed in February 2017). The evaluations were identical but the *6-min walk test* was not included in the second assessment. Thus, after excluding participants with incomplete data, a total of 70 women were finally included in the study (except for those analyses including CRF, where only 49 women were included).

The sociodemographic and clinical characteristics of the study participants are shown in Table 1.

**Table 1:** Sociodemographic and clinical characteristics (*n* = 70).

	mean	SD
Age (years)	42.5	13.9
Weight (kg)	65.4	11.2
Body mass index (kg/m2)	25.5	4.6
Disease duration (years)	13.5	10.0
SLEDAI (0–105)	0.7	1.6
Systemic Damage Index for SLE (0–46)	0.5	1.0
Current corticoicorteroids intake (yes, %)	45	64.3
Daily corticorteroids dose (mg/day)	3.8	4.9
Cumulative corticorteroids exposure (last 3 years) mg	2827.3	2704.1
Hydroxychloroquine intake (yes, %)	62	88.6
Daily hydroxychloroquine dose (mg/day)	196.5	117.2
Immunosuppressants (current intake) (yes, %)	33	47.9
Immunosuppressants (previous intake) (yes, %)	30	42.9
Organ involvement (yes, %)		
Joint involvement	50	71.4
Neurological involvement	6	8.6
Lung involvement	1	1.4
BDI-II total score (0–63)	11.8	9.0
MFI general fatigue (0–20)	13.4	4.2
Physical fitness tests		
*Flexibility*		
Back scratch dominant (cm)	2.0	9.3
Back scratch no dominant (cm)	-2.5	10.9
Back scratch mean (cm)	-0.3	9.6
*Muscle strength*		
Hand-grip strength dominant (cm)	24.1	5.5
Hand-grip strength no dominant (cm)	23.7	5.2
Hand-grip strength mean (cm)	23.9	5.2
30-sec chair stand test (repetitions)	15.0	3.2
*Cardiorespiratory fitness*(n = 49)		
6-min walk test (m)	570.8	71.8
*Criterion-referenced fitness standards (women aged 60–64)* †		
Lower body strength (yes, %)	43	60.6
Cardiorespiratory fitness (yes, %)	25	50.7
Health-related quality of life (SF-36) (0–100)		
Physical functioning	75.9	21.0
Physical role	66.5	24.7
Bodily pain	55.4	24.2
General health	42.2	19.8
Vitality	52.0	22.7
Social functioning	75.2	23.5
Role emotional	81.4	24.5
Mental health	65.3	18.6
SF-36 physical component summary	42.6	9.0
SF-36 mental component summary	46.7	10.6

BDI-II: Beck Depression Inventory second edition; BMI: Body Mass Index; MFI: Multidimensional Fatigue Inventory; SF-36: 36-item Short-Form Health Survey; SD: standard deviation; SLEDAI: systemic lupus erythematosus disease activity index

† Criterion-referenced fitness standards proposed by Rikli and Jones (2013) for women aged 60–64: lower body strength assessed by the 30-sec chair stand test = 15 repetitions; cardiorespiratory fitness assessed by the 6-min walk test = 571.5 meters.

The sociodemographic and clinical variables according to fitness level (low and high) in all fitness tests are presented in Table 2.

**Table 2:** Sociodemographic and clinical characteristic of the participants according to fitness level.

	Fexibility			Strength						CRF		
	Back Scratch Test			Handgrip			30-sec chair stand			6-min walk test		
	Low	High		Low	High		Low	High		Low	High	
	(*n =* 35)	(*n =* 35)		(*n =* 35)	(*n =* 35)		(*n =* 35)	(*n =* 35)		(*n =* 27)	(*n =* 22)	
	mean	mean	*p*	mean	mean	*p*	mean	mean	*p*	mean	mean	*p*
Age (years)	46.0 (14.2)	39.0 (12.9)	0.037	44.1 (15.8)	40.9 (11.7)	0.353	50.1 (13.1)	34.9 (10.1)	<0.001	46.8 (14.3)	34.7 (9.9)	0.002
Weight (kg)	67.3 (14.0)	63.6 (7.1)	0.173	64.1 (9.7)	66.8 (12.5)	0.311	67.4 (11.5)	63.5 (10.7)	0.153	69.6 (15.4)	62.4 (6.7)	0.049
Body mass index (kg/m2)	26.9 (5.5)	24.1 (3.1)	0.01	25.7 (4.2)	25.4 (5.1)	0.775	26.4 (4.6)	24.6 (4.5)	0.105	28.4 (5.8)	23.2 (1.6)	<0.001
Disease duration (years)	15.0 (10.1)	11.9 (9.9)	0.192	15.9 (10.7)	11.0 (8.7)	0.042	16.5 (11.0)	10.4 (8.0)	0.009	14.7 (10.0)	8.0 (6.8)	0.01
SLEDAI (0–105)	0.69 (1.5)	0.8 (1.6)	0.763	0.7 (1.5)	0.7 (1.6)	1.000	1.0 (1.8)	0.5 (1.2)	0.128	1.1 (1.9)	1.0 (1.6)	0.831
Systemic Damage Index for SLE (0–46)	0.49 (1.0)	0.5 (1.0)	0.902	0.7 (1.2)	0.2 (0.6)	0.017	0.8 (1.2)	0.2 (0.4)	0.008	0.7 (1.1)	0.2 (0.4)	0.057
Current corticoicorteroids intake (yes, %)	22 (62.9)	23 (65.7)	1.000	24 (68.6)	21 (60.0)	0.618	22 (62.9)	23 (65.7)	1.000	17 (63.0)	16 (72.7)	0.549
Daily corticorteroids dose (mg/day)	4.1 (5.6)	3.6 (4.1)	0.649	4.6 (6.0)	3.0 (3.3)	0.169	3.9 (5.6)	3.8 (4.1)	0.976	4.4 (6.1)	3.8 (3.1)	0.682
Cumulative corticorteroids exposure (last 3 years) mg	2368.2 (2477.4)	3299.9 (2879.3)	0.154	3407.6 (2956.3)	2230.0 (2310.5)	0.07	2490.0 (2651.7)	3174.5 (2752.8)	0.297	2445.1 (2593.7)	3185.4 (2784.6)	0.341
Hydroxychloroquine intake	31 (88.6)	31 (88.6)	1.000	30 (85.7)	32 (91.4)	0.71	30 (85.7)	32 (91.4)	0.71	24 (88.9)	21 (95.5)	0.617
(yes, %)												
Daily hydroxychloroquine dose (mg/day)	197.2 (122.0)	195.9 (113.9)	0.965	185.3 (113.3)	207.8 (121.5)	0.427	186.1 (120.1)	206.9 (114.9)	0.462	188.9 (111.1)	241.5 (119.5)	0.117
Immunosuppressants	15 (42.9)	18 (51.4)	0.632	19 (54.3)	14 (40.0)	0.338	13 (37.1)	20 (57.1)	0.15	10 (37.0)	11 (50.0)	0.398
(current intake) (yes, %)												
Immunosuppressants	14 (40.0)	16 (45.7)	0.809	18 (51.4)	12 (34.3)	0.227	11 (31.4)	19 (54.3)	0.09	9 (33.3)	11 (50.0)	0.26
(previous intake) (yes, %)												
Organ involvement (yes, %)												
Joint involvement	26 (74.3)	24 (68.6)	0.792	25 (71.4)	25 (71.4)	1.000	28 (80.0)	22 (62.9)	0.185	17 (63.0)	14 (63.6)	1.000
Neurological involvement	2 (5.7)	4 (11.4)	0.428	3 (8.6)	3 (8.6)	1.000	5 (14.3)	1 (2.9)	0.198	2 (7.4)	2 (9.1)	1.000
BDI-II total score (0–63)	10.60 (8.0)	12.9 (9.8)	0.284	11.0 (9.2)	12.5 (8.8)	0.484	11.4 (8.2)	12.1 (9.7)	0.742	13.9 (8.9)	12.1 (9.8)	0.515
MFI general fatigue (0–20)	13.57 (4.9)	13.3 (3.4)	0.757	13.3 (4.7)	13.5 (3.7)	0.888	13.7 (4.4)	13.1 (4.1)	0.518	14.3 (4.3)	13.3 (4.3)	0.453

Values are mean (SD) unless otherwise is indicated

An independent t test and χ2 test were used to compare variables between groups

Low and high fitness level according to the median value for flexibility (1.35 cm in the *back scratch test*), upper body strength (28.28 kg/cm^2^ in the *handgrip test*), lower body strength (15 repetitions in the *30-sec chair stand test*) and cardiorespiratory fitness (CRF; 575 m in the *6-min walk test*).

The partial correlations of physical fitness variables and HRQoL dimensions are presented in Table 3. Overall, the strength of correlations ranged from weak (r_partial_ = 0.2–0.39) to moderate (r_partial_ = 0.4–0.6). Mean upper-body flexibility (*back scratch test*) was correlated with physical functioning, social function, and PCS (r_partial_ between -0.26 and 0.28; all, *p*<0.05) and only non-dominant arm flexibility was significantly associated with those same dimensions (r_partial_ between -0.26 and 0.31; all, *p*<0.05). Mean upper-body strength (*handgrip test)* was positively correlated with physical function, physical role, bodily pain and PCS (r_partial_ between 0.27 and 0.49; all, *p*<0.05). The association of non-dominant upper body strength with HRQoL was greater compared to the dominant side for physical function (r_partial_ = 0.48 vs. r_partial_ = 0.47), physical role (r_partial_ = 0.31 vs no significance) and PCS (r_partial_ = 0.47 vs. r_partial_ = 0.42), except for bodily pain (r_partial_ = 0.26 vs. r_partial_ = 0.34). Lower-body strength (*30-sec chair stand)* was positively associated with physical function, physical role, bodily pain, and physical component (r_partial_ between 0.32 and 0.49; all, *p*<0.01). CRF (*6-min walk test*) was positively associated with physical function, bodily pain and physical component (r_partial_ between 0.39 and 0.65; all, *p*<0.01). Additional adjustments for SLEDAI, SDI and disease duration were performed, obtaining similar results (except for the non-significant association between handgrip mean strength and physical role (*p* = 0.075) and bodily pain (*p* = 0.066), and between non-dominant handgrip strength and bodily pain (*p* = 0.181) when considering this confounding variables).

**Table 3:** Partial correlations assessing the association of physical fitness components with HRQoL (*n* = 70).

* *	*Flexibility*			*Strength*				*CRF*
*HRQoL* *SF-36 dimensions*	Back scratch dominant (cm)	Back scratch non-dominant (cm)	Back scratch mean (cm)	Handgrip dominant (Kg) ^a^	Handgrip non-dominant (Kg) ^a^	Handgrip mean (Kg) ^a^	30-sec chair stand (repetitions)	6-MWT (meters) ^b^
Physical function	0.192	0.314 **	0.279 *	0.472 ***	0.478 ***	0.490 ***	0.491 ***	0.648 ***
Physical role	0.088	0.191	0.156	0.222	0.306 *	0.268 *	0.315 **	0.267
Bodily pain	0.092	0.127	0.119	0.343 **	0.259 *	0.314 **	0.396 ***	0.390 **
General health	0.159	0.185	0.186	0.074	0.148	0.108	0.022	0.014
Vitality	-0.142	-0.067	-0.107	0.067	0.135	0.1	0.204	-0.111
Social functioning	-0.215	-0.262 *	-0.259 *	0.099	0.17	0.129	0.111	0.164
Emotional role	0.02	0.089	0.063	0.047	0.062	0.055	0.162	0.108
Mental health	-0.068	-0.073	-0.076	-0.098	-0.212	-0.15	0.13	0.085
Physical component	0.191	0.286 *	0.262 *	0.424 ***	0.470 ***	0.456 ***	0.408 ***	0.478 ***
Mental component	-0.218	-0.227	-0.239	-0.188	-0.204	-0.201	0.021	-0.153

CRF: Cardiorespiratory fitness. HRQoL: Health-related quality of life. SF-36: 36-item Short-Form Health Survey. 6-MWT: 6-min walk test. Age, body mass index, depression and general fatigue were entered were entered as covariates.

^a^ *n* = 69

^b^ *n* = 49

* *p*<0.05

** *p*<0.01

*** *p*<0.001.

The regression models assessing the association of the clustered fitness score with all HRQoL dimensions are shown in Table 4. The clustered fitness score was independently associated with physical function (*B* = 17.16), bodily pain (*B* = 14.35) and the PCS (*B* = 6.02; all *p*<0.005), and there was a non-significant trend with the physical role dimension (*B* = 8.67; *p* = 0.075). Further adjustment for SLEDAI, SDI and disease duration did not alter the significance of the results.

**Table 4:** Coefficients of linear regression models assessing the association of the clustered fitness scores with HRQoL (*n =* 49).

*HRQoL* *SF-36 dimensions*	*β*	*B*	(95% CI)		*p*
**Physical function**	**0.653**	**17.159**	**10.656**	**23.662**	**<0.001**
Physical role	0.297	8.673	-0.910	18.255	0.075
**Bodily pain**	**0.445**	**14.354**	**4.584**	**24.123**	**0.005**
General health	0.036	0.821	-6.394	8.036	0.820
Vitality	-0.081	-2.313	-9.562	4.936	0.523
Social functioning	0.032	0.991	-7.415	9.397	0.813
Emotional role	0.104	2.993	-4.546	10.533	0.428
Mental health	-0.016	-0.358	-5.831	5.114	0.896
**Physical component**	**0.530**	**6.020**	**2.840**	**9.199**	**<0.001**
Mental component	-0.171	-2.324	-5.300	0.652	0.123

HRQoL: Health-related quality of life. SF-36: 36-item Short-Form Health Survey *B*, unstandardized coefficient; *β*, standardized coefficient.

Models were created using the “enter” method including age, body mass index, depression, and general fatigue as covariates. Significant associations are highlighted in bold.

**Fig 2:**
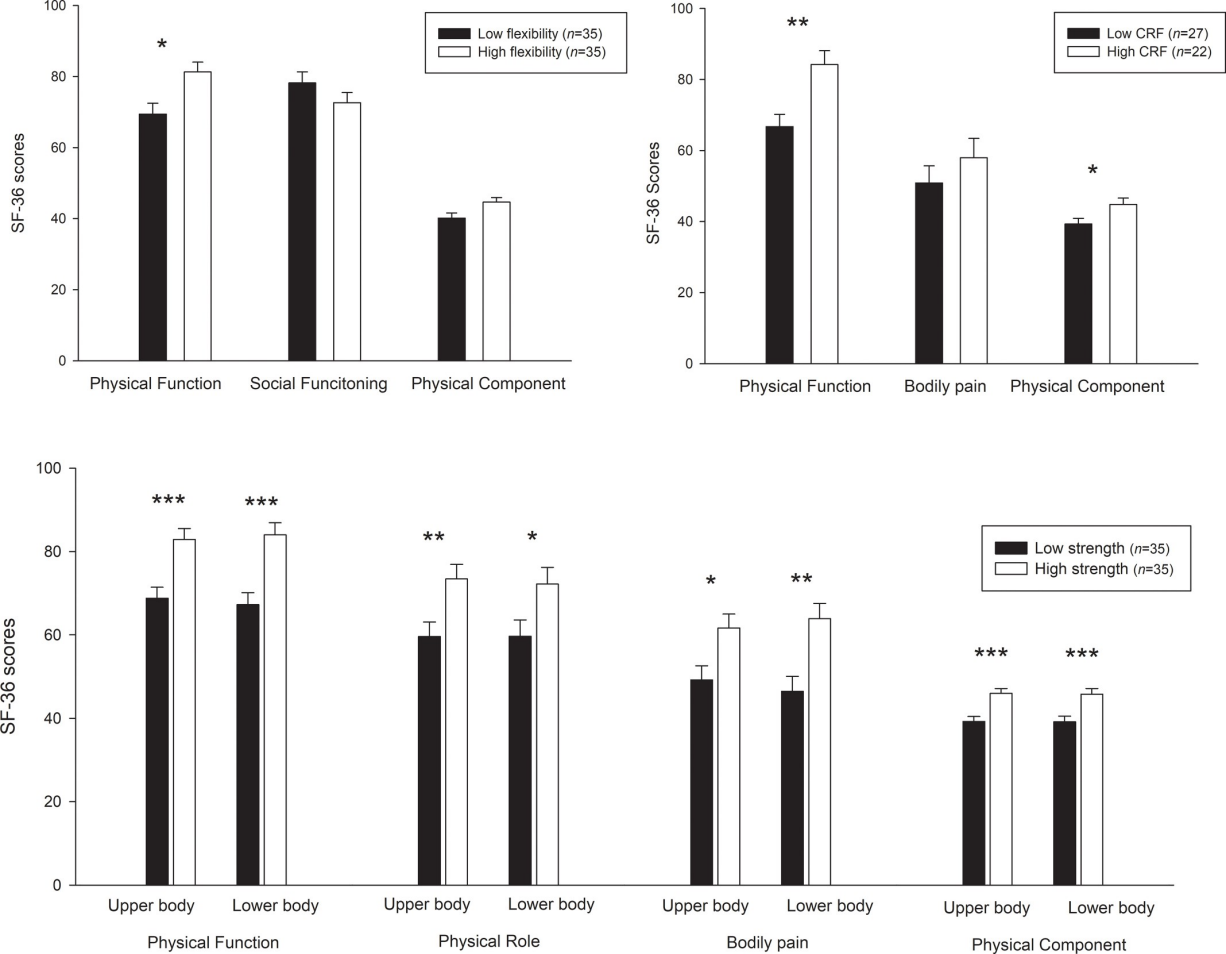
Means (95% confidence interval) of scores in the 36-item Short-Form Health Survey (SF-36) in patients with low and high fitness level according to the median value for flexibility (1.35 cm in the *back scratch test*), cardiorespiratory fitness (CRF; 575 m in the *6-min walk test*), upper body strength (28.28 kg/cm^2^ in the *handgrip test*) and lower body strength (15 repetitions in the *30-sec chair stand test*). Differences between groups were studied using analysis of covariance (ANCOVA) with age, body mass index, depression scores and general fatigue entered as covariates. *p≤0.05 **p≤0.01 *** *p*≤0.001.

Fig 2 shows the differences in specific dimensions of the SF-36 questionnaire (i.e. those that were significant related to physical fitness in previous analysis) in patients with high vs low fitness (i.e. considering the median as the cut point). Those patients with high back scratch flexibility presented higher scores in the physical function dimension (mean difference 8.75, 95%-CI = -0.01–14.46; *p* = 0.05). Those patients with a high level of CRF presented better scores in the physical function (mean difference 17.45, 95%-CI = 5.85–29.06; *p* = 0.004), and PCS (mean difference 5.47, 95%-CI = 0.11–10.83; *p* = 0.046). Participants with higher handgrip strength in the upper-body presented better scores in physical function (mean difference 14.1, 95%-CI = 6.62–21.57; *p*<0.001), physical role (mean difference 13.82, 95%-CI = 3.9–23.73; *p* = 0.007), bodily pain (mean difference 12.42, 95%-CI = 2.85–22.0; *p* = 0.012) and PCS (mean difference 6.70, 95%-CI = 3.35–10.05; *p*<0.001). Those patients with a high level of strength in the lower-body (*30-sec chair stand*) showed better scores in physical function (mean difference 16.69, 95%-CI = 7.71–25.68; *p*<0.001), physical role (mean difference 12.06, 95%-CI = -0.17–24.7; *p* = 0.05) bodily pain (mean difference 17.45, 95%-CI = 6.19–28.7; p = 0.003) and PCS (mean difference 6.57, 95%-CI = 2.39–10.75; *p* = 0.003). Further adjustment for SLEDAI, SDI and disease duration were performed obtaining similar results (except for the non-significant difference in the physical role for the 30-sec chair stand test (*p* = 0.095) and in the PCS for the 6-min walk test (*p* = 0.159) when considering these confounding variables).

## Discussion

The main findings of the present study suggest that muscle strength, and CRF are positively associated with different dimensions of HRQoL while flexibility showed contradictory results. The clustered fitness score was consistently associated with better scores in the physical function, bodily pain, and PCS. Patients with a high fitness level in all components presented better scores in those dimensions of HRQoL associated with physical function. Overall, there was no association of physical fitness with any of the mental-related dimensions of HRQoL. These results warrant the implementation of future prospective research and clinical trials to understand the prognostic value of fitness and the extent to which increasing fitness through exercise might be linked to improvements in HRQoL.

The reduced fitness level of the patients in the present study is in agreement with prior research [4–7]. We additionally observed that a 60% or less of our participants (aged, on average, 42.1 years), reached the level of lower-body strength and CRF proposed for women aged 60–64 to maintain physical independence [27]. These findings suggest that a considerable number of relatively young patients with SLE are already disabled or at risk of losing the physical capacity needed for daily living during their later life [27], stressing the importance of promoting exercise interventions that increase fitness level. Consistently, in further comparisons between groups of high and low fitness (according to the median value of the group) we detected mean significant differences in HRQoL of at least 12 points in single dimensions and 6.5 points in the PCS. Different thresholds have been proposed for minimal clinically important changes in the SF-36 in SLE, ranging from 5 to 10 points difference in single dimensions [41] and a minimum difference of 2.1 points for the PCS[42]. Considering the reduced fitness level used as cut-point for the comparisons, it could be argued that even moving from an extremely low to a relative low fitness level could be potentially related to relevant changes in HRQoL, yet this hypothesis needs to be confirmed in future intervention studies.

Cardiorespiratory fitness seems to be the fitness component that presented the strongest association with HRQoL, more specifically with physical function, bodily pain and PCS, beyond the non-significant association with the physical role dimension. Two previous studies examined the relationship of CRF with HRQoL in SLE [7,28]. Tench et al. [7] showed that a better peak aerobic capacity was associated with better physical function dimension. Additionally, those patients able to perform the treadmill test at a greater speed reported better physical function, physical role, and bodily pain [7]. Despite the use of different tools to assess CRF, our results generally concur with these previous findings. In other study, CRF assessed with the *6-min walk test* was positively associated with physical function, social function, emotional role, and mental health in patients with SLE. As opposed to Balsamo et al.[28] and our initial hypothesis, we observed no association of CRF with mental dimensions of HRQoL. It must be noted that, contrary to the study of that Balsamo et al.[28], our analyses were adjusted for different psychosocial and clinical variables. Our results, however, are in agreement with studies in other conditions that observed associations of CRF with physical health rather than mental health [43]. Taking together our findings and those from previous research in SLE, it seems that there is a link between CRF and HRQoL, which is more consistent across the physical dimensions and that needs to be further explored for mental components.

Another major finding is that muscle strength was the component that presented correlations and significant differences in more dimensions of HRQoL. These associations were also roughly equal between dominant, non-dominant sides and mean values. However, for physical role the association with dominant grip strength was non-significant (*p* = 0.06). We also observed a slightly greater association of the mean strength which is in line with recent evidence stressing the relevance of including a relative measure of strength that considers both hands as a better indicator of health in comparison to dominant hand [44]. In patients with SLE, a positive association of dynamic maximum muscle strength (upper and lower limbs) with physical function and emotional role has been described [26]. Furthermore, Andrews et al. [29] reported that lower-body muscle strength (assessed by peak isokinetic knee torque and chair-stand time) was positively associated with physical functioning. This association was additionally confirmed in longitudinal research were a reduced lower extremity muscle strength predicted declines in physical function [45]. Our results not only corroborate prior research but also extend the findings to non-previously explored dimensions of HRQoL. Upper and lower extremity muscle strength were additionally associated with physical role, bodily pain and the PCS in this study which is in concordance with previous studies in other rheumatologic populations [22].

Literature regarding flexibility in SLE is merely descriptive [5,26] and no implications for health of this component in isolation has been stablished either in observational or intervention studies. In the present study, we found contradictory results: while there was a positive association of flexibility with physical function and physical component, there was a negative association with social function. This finding contrasts with the general positive association between all fitness components and HRQoL [46], and also with the benefits for mental health of flexibility interventions [47]. It is noteworthy, however, that flexibility showed the weakest correlations among all fitness components and that we did not find any differences in social dimension between groups of high and low flexibility. Also, the non-dominant limb presented stronger correlations compared to the dominant side. It is difficult to elucidate the nature of these findings as there is a lack of previous studies assessing how flexibility of each side of the body is related to health. For the descriptive values in our participants we could observe a 4.5 mean difference between dominant and non-dominant flexibility which is in line with prior evidence showing a reduced mobility in upper-body non-dominant side, probably related to usage [48]. It could be hypothesized that the relationship between flexibility and HRQoL may not be uniform across the whole spectrum of values but only relevant until the minimum level of mobility required for daily living activities from which greater flexibility would not represent greater benefits in HRQoL. Nevertheless, these exploratory findings need to be assessed in detail in future studies. Despite of these results, flexibility has recently gained attention due to its potential role in cardiometabolic health [49] and pain reduction in SLE [19] and its inclusion in exercise programs is currently encouraged [50]. Future interventions studies are needed to examine these contradictory findings regarding flexibility and to determine the adequate regimes of intensity, frequency, and progression for possible health benefits in patients with SLE.

Different hypothesis may explain the associations found between fitness and the HRQoL dimensions (mainly physical function, bodily pain and the PCS). Increasing fitness could improve the ability to perform daily living tasks assessed in physical function dimension [31] that typically requires a combination of CRF, strength and flexibility. However, as proposed by Balsamo et al. [28], it is also plausible that the relationship worked bidirectionally, with better HRQoL predicting better physical fitness. Muscle strength and CRF were the main contributors for the associations found between fitness and bodily pain, which is a frequently reported symptom in SLE [51]. Among the various causes of pain in SLE [51], exercise could act on reducing inflammation [52] or through an improvement of muscle oxygenation [53] that reduces peripheral and central sensitization[54,55], as suggested for other rheumatic conditions. Nonetheless, the scarce evidence in SLE regarding the changes on pain after exercise is inconclusive [15–19], and not necessarily related to changes in fitness [18,19]. Importantly, our findings also showed that the relationships found between fitness and HRQoL are independent of psychosocial and clinical aspects such depression, fatigue, or IMC, limiting the explanatory capacity of these frequent conditions concomitant to SLE. We must note that the correlations found between fitness and HRQoL ranged from weak to moderate. However, considering the multiple factors affecting HRQoL, the possible explanatory capacity of single variables is low and, therefore, these weak-moderate correlations were somehow expected and still of relevance. Also, we found a lack of association between physical fitness and mental domains. We hypothesized that physical fitness may be a greater determinant for the limitations assessed in the physical component while other factors such age [56], disease activity or duration [57,58] may be closer to mental health in SLE. Our findings in a relatively young with clinical stability sample would support this idea as we observed better scores for mental domains compared to physical domains when the values of the SF-36 questionnaire were compared to reference values of the Spanish population [59].

This study has limitations. The cross-sectional design does not allow establishing causal relationships. The sample size was relatively small and included patients with low disease activity. Consequently, our results may not be generalizable to the entire SLE population. In addition, the comparisons between “fit” and “unfit” individuals were performed according to an arbitrary cut point (median value). Because the Senior Fitness Test for older adults was used, our results in an adult population are not completely comparable with the reference standards. In future research, it would be of interest to assess other fitness components (lower body flexibility or agility) and the role of other clinical variables such us disease activity, concomitant therapy, or the numbers of the used drugs. Our findings might have implications for the development of exercise-based intervention studies, especially in those aimed to enhance specific components of physical fitness in women with SLE.

## Conclusion

In summary, higher muscle strength, CRF, and global physical fitness were positively associated with different dimensions of HRQoL. The role of flexibility was contradictory and deserves further research. Patients with high fitness presented better scores in HRQoL, especially in those dimensions associated with physical health. Overall, there was no association of physical fitness with any of the mental-related dimensions of HRQoL. Future research is needed to understand the prognostic value of fitness and the extent to which increasing fitness through exercise might be linked to improvements in HRQoL.

## Supporting information

S1 File Database. (XLSX)
